# The Impact of Purebred Zebu Breeds on Growth Performance and Carcass Characteristics

**DOI:** 10.3390/ani15203024

**Published:** 2025-10-17

**Authors:** Jonatã Henrique Rezende-de-Souza, Nara Regina Brandão Cônsolo, Leonardo de Oliveira Fernandes, Lauro Fraga Almeida, Giovana Alcantara Maciel, Ninive Jhors Carneiro Reis, Anders H. Karlsson, Sergio Bertelli Pflanzer

**Affiliations:** 1 Department of Engineering and Food Technology, School of Food Engineering, University of Campinas, Campinas 13083-862, Brazil; 2 Department of Nutrition and Animal Production, School of Veterinary Medicine and Animal Science, University of São Paulo, Pirassununga 13635-900, Brazil; nara.consolo@usp.br; 3 Empresa de Pesquisa Agropecuária de Minas Gerais, Uberaba 38060-040, Brazil; leonardo@epamig.br; 4 Associação Brasileira dos Criadores de Zebu, Uberaba 38022-330, Brazil; tecnico097@abcz.org.br (L.F.A.); ninive@abcz.org.br (N.J.C.R.); 5 Empresa Brasileira de Pesquisa Agropecuária, Planaltina 73310-970, Brazil; giovana.maciel@embrapa.br; 6 Department of Applied Animal Science and Welfare, Swedish University of Agricultural Sciences, 53231 Skara, Sweden; anders.h.karlsson@slu.se

**Keywords:** *Bos indicus*, Brahman, Guzerat, Sindhi, Tabapua, ultrasound

## Abstract

This study aimed to evaluate the growth performance and carcass characteristics of young intact male purebred origin Zebu cattle of four breeds: Brahman (n = 17), Guzerat (n = 25), Sindhi (n = 23), and Tabapua (n = 41). A total of 106 animals were weaned at 8–9 months, reared together on *Urochloa brizantha* cv. Paiaguás pasture for 280 days, and subsequently finished in an intensive feedlot for 130 days. Animal development was assessed through serial weighing, carcass ultrasound measurements, and feed intake monitoring. Post-slaughter, hot and cold carcass traits were registered. Sindhi cattle exhibited lower birth and final body weights, averaging 508.6 kg, compared to 602.7–628.9 kg for the other breeds (*p* < 0.05), reflecting their smaller body structure and lower feed intake (18.00 kg/day vs. 21.27–22.14 kg/day for the other breeds). However, despite their reduced dry matter intake (10.17 kg/day), Sindhi showed one of the most favorable feed conversion ratios (9.08), comparable to Guzerat (9.27), both more efficient than Brahman (7.42) and Tabapua (8.05) (*p* < 0.05). Brahman animals had the greatest rib eye area (REA) during both pasture (75.43 cm^2^) and at finishing (92.31 cm^2^). Hot carcass weight ranged from 333.4–353.9 kg for Brahman, Guzerat and Tabapua, while for Sindhi, the weight was 285.9 kg. However, Sindhi showed favorable carcass yield and usable meat content, despite reduced body development. Loin and rump fat thickness showed little variation across breeds, and marbling scores did not differ. These findings demonstrate that while heavier breeds like Brahman excel in muscle deposition and carcass weight, lighter breeds like Sindhi are efficient in converting feed into meat, offering competitive carcass yields. The results reinforce the value of breed-specific management strategies to optimize beef production and support Brazil’s competitiveness in global meat markets.

## Simple Summary

Brazil plays a significant role in global beef production, holding the world’s largest commercial cattle herd. This study provides practical insights for improving beef production in Brazil and other tropical countries. Evaluating underrepresented Zebu breeds reveals opportunities for genetic selection targeting carcass yield, production efficiency, and meat quality. Distinct patterns of growth, feed intake, and performance indicators among breeds can enable more precise planning of slaughter and feed management, reducing environmental impact and production costs. Additionally, optimizing breed use enhances meat quality consistency for consumers. These findings can help diversify genetic resources beyond Nellore, promote sustainable beef systems, and reinforce Brazil’s global competitiveness as the largest exporter of tropical beef.

## 1. Introduction

The global beef market is highly competitive, with increasing demand for differentiated and high-value products. Brazil plays a significant role in global beef production, holding the world’s largest commercial cattle herd, with 197.2 million cattle and buffalo in 2023 [1]. The country is also the second-largest beef producer and the leading global exporter, supplying key markets such as China, the United States, Hong Kong, Chile, and the European Union [1]. This market prioritizes price efficiency and sustainable production practices [1,2,3]. To meet these demands, the Brazilian beef industry has evolved, investing in technology, efficient management, animal welfare, and genetic improvement to enhance meat quality and strengthen its market position [1,4,5,6,7].

Animal genetics plays a fundamental role in beef production. In Brazil, approximately 80% of beef comes from Zebu cattle, mainly Nellore and crossbred animals resulting from Nellore crossed with other Zebu or Taurine breeds. These animals are well adapted to tropical climates, showing resilience to parasites and endemic diseases, while also delivering favorable carcass yields and usable meat content, contributing to global market competitiveness [8]. Beyond Brazil, other major beef-exporting countries in tropical environments, such as Australia, India, Paraguay, and Mexico, also keep Zebu cattle in their herds. This confirms the global importance of studying *Bos indicus* genetics, as Zebu beef is widespread worldwide. Additionally, climate change has intensified global warming trends [9], further reinforcing the need for studies on Zebu breeds, given their greater heat tolerance compared to Taurine (*Bos taurus*) cattle.

Historically, Brazilian beef production was based on slaughtering intact males over 36 months of age [1,10]. However, this trend has shifted, with only 11.72% of slaughtered males now exceeding 36 months [1]. Reducing slaughter age, combined with efficient nutritional management, has contributed to improvements in meat quality [6,7,11,12]. Additionally, other Zebu breeds, such as Brahman, Guzerat, Sindhi, and Tabapua, have gained attention in Brazilian beef production [13]. However, some of these breeds remain underrepresented in animal and meat science research due to their lower prevalence in commercial production, when compared to the Nellore.

Genetic selection has been widely used to enhance productivity and meat quality [14,15]. Rosse et al. [14] sequenced the Guzerat genome, identifying key genes associated with reproductive performance, disease resistance, heat tolerance, and intramuscular fat deposition. To preserve and optimize these genetic traits across generations, Santana et al. [13] proposed breeding strategies based on pedigree and herd size. For Guzerat, Sindhi, and Tabapua cattle, cross-herd breeding was recommended to enhance genetic exchange and minimize population subdivision, whereas for Brahman, maintaining within-herd genetic diversity was advised.

Genetic improvements through selection and strategic crossbreeding, combined with efficient management, can optimize desirable traits such as accelerated growth, increased muscle deposition, and appropriate fat coverage. This process is significantly enhanced by the use of purebred animals, which allow for a more accurate identification and selection of traits of interest due to their genetic uniformity. Purebreds offer greater consistency and predictability in the expression of heritable characteristics, facilitating the development of effective breeding programs and reinforcing their importance in both scientific research and genetic improvement strategies.

Moura et al. [16] demonstrated that purebred Nellore animals exhibited lower rates of heat production, thermal storage, and skin evaporation compared to F1 crossbred animals (Nellore × Angus), indicating superior thermoregulatory efficiency, an advantageous trait for production in tropical environments. These findings reinforce the importance of including purebred animals in genetic studies and breeding strategies aimed at enhancing adaptation and performance. Understanding the genetic basis of beef production and its impact on meat quality is essential for developing more efficient and sustainable production systems. Therefore, this study aimed to evaluate the influence of purebred Zebu cattle on production performance (serial weighing, carcass ultrasound measurements, and feed intake monitoring) and carcass traits (fat measurements and carcass yield).

## 2. Materials and Methods

### 2.1. Animals, Diet, and Production System

The animal study protocol was approved by the Institutional Review Board of EPAMIG (protocol code 02/2020). The experiment was conducted at the Orestes Prata Tibery Júnior Experimental Farm, owned by the Associação Brasileira dos Criadores de Zebu—ABCZ (in English: Brazilian Association of Zebu Breeders), on a 27-hectare area located in Uberaba, 476 km from Belo Horizonte, Minas Gerais, Brazil (−19.71, −47.96). The region’s climate is typically tropical, characterized by hot and humid summers and dry and mild winters. For this experiment, intact male animals (uncastrated), purebred origin (PO), were used, and all production activities followed commercial practices. After slaughter, the meat was sold to the market as usual.

**Figure 1:**
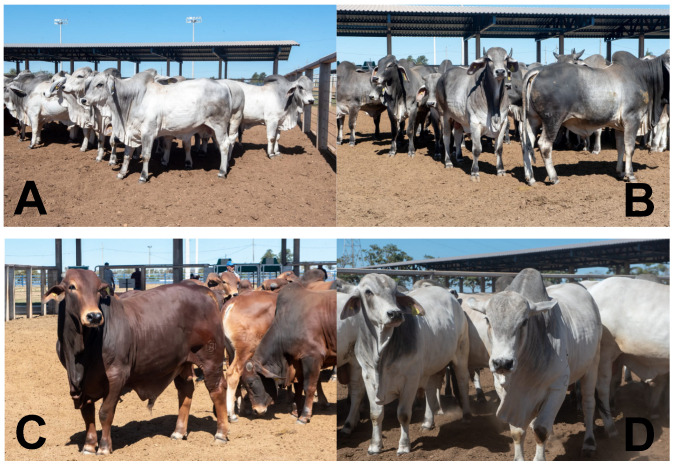
Representation of breeds Brahman (**A**), Guzerat (**B**), Sindhi (**C**), and Tabapua (**D**); these images were recorded at the end of the fattening period.

Male calves born between September and October 2020 had a suckling period of 8–9 months, after which 106 animals were selected for the study, distributed across four breeds: 17 Brahman, 25 Guzerat, 23 Sindhi, and 41 Tabapua (Figure 1). Selection was based on the EPMURAS method (Table 1), which evaluates many production and development attributes of live animals between weaning to the initial grazing system [17,18], and is a widely used method in Brazilian cattle production. Animals showing favorable results for different production and development indicators were included in the study (Table 2).

The pasture-feeding phase began on 8 June 2021, with animals exhibiting an average live weight of 222.9 kg. Initially, the animals were fed *Urochloa brizantha* cv. Paiaguás grass in a 20.59-hectare area for a total of 280 days, divided into 140 days during the dry season and another 140 days during the rainy season. Both periods were managed using rotational grazing across eight paddocks. Animals were introduced into the paddock when the grass height reached 45 cm and removed when it decreased to 25 cm. Soil liming and fertilization were performed, applying 120 kg of N (nitrogen), 80 kg of P_2_O_5_ (phosphorus pentoxide), and 110 kg of K_2_O (potassium oxide).

During the dry season, from June to October 2021 (140 days), animals were supplemented with a protein-energy supplement in addition to the pasture, provided as 0.5% of body weight on a dry matter basis. This supplement contained 24% crude protein (CP) and 64.4% total digestible nutrients (TDN). Complementarily, corn silage was offered at 1% of the animals’ body weight. During the rainy season, from late October to mid-March 2022 (140 days), the diet was based on pasture supplemented with a protein-energy concentrate equivalent to 0.4% of body weight. This supplement contained 12% CP and 67% TDN. Details regarding the supplemental feed are provided in Supplementary Table S1, while the composition of the pasture during the dry and rainy seasons, as well as the corn silage, is presented in Table 3. Throughout the pasture-feeding period, animals had free access to water, supplied via electronic drinkers integrated with scales, allowing for automatic recording of water intake data.

Following the pasture-feeding period, animals were confined for 130 days, from March to late July 2022. The animals were housed in seven pens of 30 m^2^ each, with distribution based on body weight to minimize competition for feed. All pens provided natural ventilation and lighting, along with artificial shade at a density of 4 m^2^ per animal. The diet consisted of 60% concentrate (concentrate Premix—16% CP; 74.0% TDN), 33% corn silage, and 7% hay. Fresh water was provided ad libitum throughout the day, and individual water intake was recorded using electronic bunks integrated with weighing systems. The diet was provided using integrated electronic feed bunks, three times a day (07:00, 10:00, and 15:00 h.), with a composition of 14% CP and 73.4% TDN. Daily feed refusals were maintained between 5% and 10% of the amount offered, with daily adjustments to ensure ad libitum intake.

### 2.2. Growth Performance and Feed Efficiency

During both the pasture and feedlot periods, animals were weighed every 28 days to record individual development. Using the recorded weights, average daily weight gain and final weight during each period were calculated using Equations (1) and (2), respectively. Only during the feedlot period was individual feed intake measured daily using automated feeders equipped with weighing cells (AF-4.69 troughs, Ponta, Brazil). This enabled the determination of average feed intake. Feed conversion and feed efficiency were also calculated using the data on dry matter intake (kg) and daily weight gain (kg) during the feedlot period, as described in Equations (3) and (4).

(1) $$Daily\;gain\;(kg/day)=\frac{Final\;weight\;of\;the\;period\;(kg)-Initial\;weight\;of\;the\;period\;(kg)}{Duration\;of\;the\;period\;(days)}$$

(2) Total gain (kg)=Final weight period (kg)−Initial weight period (kg)

(3) $$Feed\;conversion=\frac{Dry\;matter\;intake\;(kg)}{Daily\;weight\;gain\;(kg/day)}$$

(4) $$Feed\;efficiency=\frac{Daily\;weight\;gain\;(kg/day)}{Dry\;matter\;intake\;(kg)}$$

### 2.3. Muscular and Carcass Development

Individual ultrasound measurements of the animals were performed at the end of both the pasture and feedlot periods to monitor muscle development and fat deposition. All measurements were conducted and evaluated by the same technician to minimize error variation, using 500V1 ultrasound equipment (Aloka, Musashino, Japan) with a 3.5 MHz linear transducer, 17.2 cm in length (Aloka, Musashino, Japan). The procedure was based on the methodology described by Avilés et al. [19]. The cattle were restrained, and images were obtained without the need for hair removal. For this purpose, vegetable oil was applied to improve contact between the skin and the probe. The evaluated measurements included the rib eye area (REA) between the 12th and 13th vertebrae, subcutaneous fat thickness, also measured between the 12th and 13th vertebrae, and rump fat thickness measured between the *Biceps femoris* and *Gluteus medius* muscles.

### 2.4. Slaughter and Carcasses Evaluations

Slaughter was conducted after 130 days of feedlot period, when the animals were approximately 22 to 24 months of age, in a slaughterhouse located in the State of São Paulo, Brazil, certified by the Brazilian Federal Inspection Service. Prior to slaughter, the animals were weighed after fasting for 12 h to record their live body weight. Slaughter was performed following the guidelines of the Industrial and Sanitary Inspection Regulation for Animal-Origin Products [20]. After skin removal and evisceration stages, hot carcass evaluations were carried out, and after cooling the carcasses for at least 30 h, cold carcass evaluations were performed.

The half-carcasses were weighed to determine the hot carcass weight. Additionally, the distribution of carcass fat was evaluated, classifying the carcasses into the following categories: absent, when fat coverage is non-existent or very thin (<1 mm thickness); Slight, when the visual presence of muscle exceeds that of fat coverage (1–3 mm); medium, when the muscles are almost always covered with fat except in the hindquarter region (4–6 mm); uniform, when the muscles are covered with fat but only partially covered in the hindquarter region (7–10 mm); and excessive, when the entire carcass, including the thoracic cavity, is covered with fat (>10 mm). These hot carcass evaluations were based on the Brazilian Beef Carcass Classification System [21].

On cold carcasses, a cut was made between the 12th and 13th thoracic vertebrae for evaluation. Standards described in the USDA Quality Grade were used to assess skeletal and lean maturity, both scored from A00 to E00 [22,23]. Marbling was also evaluated using the USDA Quality Grade standards, as detailed in the Meat Evaluation Handbook, with scores ranging from Practically Devoid to Abundant. Subcutaneous fat thickness was measured perpendicularly to the external surface of the carcass using a digital caliper [22]. To measure the rib eye area (REA), a green pattern measuring 2 × 5 cm was placed in the lateral region of the *Longissimus lumborum* muscle, followed by capturing a photograph. The REA was then determined in the photo, through calculations using the pattern size, with the help of AxioVision Rel.4.8 Software.

Using some of this information, it was possible to determine carcass yield and total usable meat based on Equations (5) and (6), respectively, where HCW represents hot carcass weight, LW is the live weight of the animal before slaughter (after fasting), SFT is subcutaneous fat thickness, and REA is rib eye area [24], with modifications.

(5) $$Carcass\;yield\left(\%\right)=\frac{Hot\;carcass\;weight\;(kg)}{Live\;weight\;(kg)}\times 100$$

(6) $$Total\;usable\;meat\left(\%\right)=75-\left(0.02\times HCW\right)-\left(0.489\times SFT\right)+(0.0119\times REA)$$

### 2.5. Data Analyses

The statistical procedures were performed using the Statistica software package (StatSoft, Inc., 2011; version 10.0, Tulsa, OK, USA). The data were analyzed using one-way ANOVA, preceded by verification of its assumptions. To assess normality, the Shapiro–Wilk test was applied to each breed within each dependent variable, with normality assumed when *p* > 0.05. Homoscedasticity was subsequently verified using Levene’s test, with variables considered homogeneous when *p* > 0.05. For variables in which at least one ANOVA assumption was violated, the nonparametric Kruskal–Wallis test was applied. Nonparametric variables included birth weight, fattening period parameters such as water intake and feed conversion, as well as ultrasound measurements like 12th rib fat thickness and rump fat thickness. Additionally, carcass traits such as skeletal maturity, lean maturity, marbling, fatness score, carcass yield, total usable meat, 12th rib fat thickness, and rib eye area were also evaluated with nonparametric tests. All other variables met the assumptions of normality and homoscedasticity and were analyzed using one-way ANOVA, followed by Tukey’s test for post hoc comparisons (*p* < 0.05).

## 3. Results

### 3.1. Growth Performance and Feed Efficiency

The growth performance and feed efficiency indicators are presented in Table 4. Birth weight was significantly lower for Sindhi animals (27.52 kg) compared to the other breeds (34.38–35.65 kg). During the initial life stage, characterized by the suckling period (8–9 months), Sindhi animals had the lowest final weight for this period (202.39 kg), as well as the lowest daily and total weight gains during suckling (0.68 kg/day and 174.87 kg, respectively), when compared to Guzerat and Tabapua animals, with no significant difference relative to Brahman.

A similar trend was observed during the first grazing period, which corresponded to the dry season (Table 4). At the end of this period, Guzerat and Tabapua animals weighed 317.64 kg and 313.07 kg, respectively, while the Sindhi animals had the lowest weight, 276.48 kg (*p* < 0.05). However, daily and total weight gains during this period were not affected by breed (*p* > 0.05). In the second grazing period, characterized by the rainy season, Sindhi animals reported significantly the lowest final weight (359.48 kg), as well as the lowest daily and total weight gains (0.59 kg/day and 83.0 kg, respectively), when compared to the other breeds (Table 4). When combining the dry and rainy grazing periods, the trend persisted, with Sindhi animals showing the lowest final weights and lowest weight gains (*p* < 0.05).

After 280 days on pasture, the animals were finished in feedlot for 130 days. At the end of this period, the lower weight trend for Sindhi animals continued (Table 4). Brahman, Guzerat, and Tabapua breeds had final weights ranging from 602.72 kg to 628.86 kg, which were higher than those of the Sindhi animals (*p* < 0.05), with Brahman and Tabapua also displaying the highest daily and total weight gains, followed by Guzerat, while Sindhi animals had the lowest values for these indicators (*p* < 0.05).

Like growth performance indicators, Sindhi animals also showed the lowest values for feed efficiency indicators overall (Table 5). Feed intake was 18 kg/day for Sindhi animals, significantly lower than the values above 21 kg/day reported for the other breeds. Consequently, dry matter intake was also lower for Sindhi (*p* < 0.05). Brahman and Tabapua breeds reported the lowest feed conversion ratios, while Brahman achieved the highest feed efficiency ratio (*p* < 0.05).

### 3.2. Muscular and Carcass Development

Ultrasound measurements in live animals are shown in Table 6. Brahman animals had the largest rib eye area (REA) during both the grazing and feedlot periods (*p* < 0.05). During the grazing period, the Brahman had an REA of 75.43 cm^2^, while the other breeds presented values close to 70 cm^2^. In feedlot, Brahman maintained the highest REA (92.31 cm^2^), significantly greater than Guzerat and Sindhi, both with approximately 82 cm^2^. Fat thickness over the loin (12th rib) was not affected by breed during the grazing period (*p* > 0.05), varying between 2.21 and 2.38 mm. However, during feedlot period, fat thickness was significantly higher in Tabapua compared to Guzerat (5.22 mm vs. 4.31 mm, respectively). Regarding rump fat thickness, Brahman animals had significantly greater values (4.45 mm) compared to Guzerat and Tabapua (3.75 mm and 3.89 mm, respectively) during the grazing period, but no significant breed effect was observed during feedlot, with values ranging from 6.67 to 7.23 mm.

### 3.3. Carcass Characteristics

**Figure 2:**
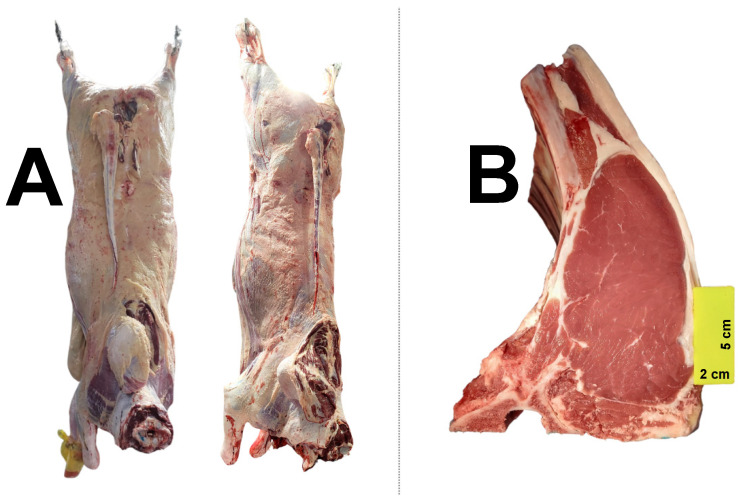
Visual representation of hot carcasses (**A**) and rib eye area (**B**).

Fatness scores and marbling content did not differ significantly among breeds (Table 7), and a visual representation of the carcasses is provided in Figure 2. Consistent with the growth performance data presented earlier in Table 4, hot carcass weight was significantly lower for Sindhi animals, at 285.85 kg, while for the other breeds, the hot carcass weight ranged from 333.36 kg to 353.88 kg (Table 7). However, the Brahman breed exhibited the lowest carcass yield at 54.40% (Table 7). The total usable meat yield was significantly lower for Tabapua compared to Brahman and Sindhi (Table 7), although the variation among breeds was small, ranging from 75.19% to 76.46%.

Fat thickness and REA were also influenced by breeds (Table 7). Tabapua animals had the greatest fat thickness, 4.45 mm, while Guzerat had 3.28 mm, the lowest value among all breeds (*p* < 0.05). Brahman animals had the largest REA (85.35 cm^2^), reflecting greater muscle deposition compared to Sindhi (73.28 cm^2^), which had significantly smaller values (Table 7). Conversely, fat thickness and REA values measured in cold carcasses (Table 7) were lower than those observed by ultrasound during the feedlot period (Table 6). However, the trends were consistent: the highest values were recorded for the same breeds in both assessments.

## 4. Discussion

### 4.1. Growth Performance and Feed Efficiency

The variation in weight across different time points for the evaluated breeds reflects phenotypic variations and genetic selection. Birth weights ranged from 27.52 kg for Sindhi to values above 34 kg for the other breeds. These values are consistent with birth weights reported for different breeds in previous studies [25,26,27]. Lamartine-Paiva et al. [28] reported a mean birth weight of 25.35 kg for a group of 20 Sindhi bulls born in the northeastern region of Brazil, which is slightly lower compared to the Sindhi animals in this study. The lower average birth weight for Sindhi could reflect its reduced intrauterine growth potential compared to other Zebu breeds. In contrast, higher birth weights for Brahman, Guzerat, and Tabapua may be correlated with a larger body size of these breeds.

During the first 140 days on pasture (dry season), breed did not influence daily or total weight gains, but there was a significant variation in final weight among the breeds during this period (Table 4). However, even with this variation, the weight trends were reflective of initial values, with Brahman showing intermediate gains, while Guzerat and Tabapua achieved significantly higher values, and Sindhi showed the lowest performance. During the rainy season (the final 140 days on pasture), daily and total weight gains, as well as final weight for this period, were significantly lower for Sindhi, showing a breed difference, with smaller muscle size from birth until the final stages of pasture period. These trends reflect the inherent characteristics of Sindhi cattle. As a dual-purpose breed, genetic improvement programs in Brazil have primarily focused on enhancing feed conversion efficiency for both milk and meat production, thereby reducing feeding costs. In contrast, for other breeds such as Brahman, one of the main breeding objectives is weight gain and fat deposition for beef production.

A similar trend was observed when analyzing daily and total weight gains throughout the entire pasture feeding period (280 days). However, during feedlot, daily and total weight gains were even more pronounced in Brahman and Tabapua compared to the other breeds, reflecting greater efficiency in utilizing the concentrated diet during this period. Once again, Sindhi demonstrated lower values for these weighing indicators, with the lowest daily and total gains, reinforcing the characteristics of their racial pattern of smaller muscle size compared to the other breeds evaluated. The observed developmental patterns of the animals in this study are thus an intrinsic reflection of their genetic composition. Previous studies have shown moderate to high correlations between birth weight and developmental weight for different Zebu cattle breeds [29,30], a trend also observed in this study.

Slaughter weights, achieved after the feedlot period, exceeded 600 kg for the Brahman, Guzerat, and Tabapua groups, differing significantly from Sindhi, which had a slaughter weight of 508.6 kg. These results surpass the slaughter weights reported for Nellore, the predominant Zebu breed in Brazil, as well as for other less commonly produced breeds. Nellore animals were pasture-fed until 20 months of age, finished in three different ways: two semi-feedlot treatments with pasture for 40 or 80 days, and full feedlot. Their slaughter weights were 475.33 kg, 460.67 kg, and 534.92 kg, respectively [7]. Nellore finishing in feedlot for 112 days with a diet consisting of 74.3% total digestible nutrients and 15% crude protein resulted in a slaughter weight of 475.4 kg at 34 months of age [4]. Additionally, the slaughter weights observed in this study were higher than those reported in previous studies for other purebred or crossbred cattle. Ribeiro et al. [31] evaluated castrated males raised on pasture and slaughtered at just over three years of age, with slaughter weights of 474.0 kg for Nellore and 469.7 kg for ½ Guzerat × ½ Nellore, both significantly lower than the 498.8 kg recorded for ½ Brahman × ½ Nellore. Brahman cattle fed on pasture and slaughtered at 34–35 months of age had slaughter weights of 486.4 kg and 509.6 kg in nervous and calm animals, respectively [32]. Guzerat bulls, uncastrated and aged 30–34 months, finished in an intensive system with 50–80% concentrate in the diet, achieved an average slaughter weight of 578.1 kg and 555.4 kg, respectively [5]. Thus, it can be emphasized that not only genetics influences weight gain, but also the use of an efficient production system between breeding, pasture rearing, and finishing is crucial for promoting greater total weight gain.

The animals in this study achieved growth performance characteristics close to those of taurine animals raised in different systems, slaughtered around 27 months of age [33]. The results were also similar to those reported for 15 different breeds of European young bulls, which were slaughtered at around 15 months of age, with slaughter weight variation ranging from 378.4 kg for Jersey to 634.0 kg for Charolais, with Limousin weighing 565.4 kg, and Aberdeen Angus, the most popular taurine breed, reaching a slaughter weight of 597.7 kg [34]. This further reinforces the importance of utilizing an efficient animal management system, coupled with the selection of animals with good growth potential. The breeds used in this study highlight the importance of intensifying meat production in Brazil, contributing even more to the Brazilian production system.

Overall, it is evident that throughout the development of the animals, Sindhi consistently exhibited the lowest values not only in weight measurements but also in daily and total weight gain rates for the different production periods. These results are associated with the breed’s characteristics and are aligned with its lower feed intake. The feed conversion rates observed in the different racial groups analyzed in this study were higher than those reported by Homem Junior et al. [5], which ranged from 6.1% to 7.1% for Guzerat animals of early and late body types fed with diets containing 50% or 80% concentrate. These rates were also higher than those reported for Nellore (6.04%), Aberdeen Angus × Nellore (5.42%), and Canchim × Nellore (5.54%), while feed efficiency was 0.172%, 1.188%, and 1.183%, respectively [6]. These values were higher than those reported for the genetic groups in this study (0.109–0.136%). In contrast, despite demonstrating better feed conversion for Guzerat and Sindhi, Brahman animals exhibited greater feed efficiency, corroborating their prominence in weight gain and the subsequent ability to convert feed into live weight during feedlot. Therefore, the results obtained clearly highlight differences in growth performance and feed efficiency among the evaluated breeds, reflecting the influence of genetics on these productive indicators.

### 4.2. Ultrasound Measurements

The Brahman animals exhibited the highest values for rib eye area (REA) in both evaluation periods, namely during pasture feeding (75.43 cm^2^) and during feedlot (92.31 cm^2^), while the other racial groups had values ranging from 69.17 to 70.44 cm^2^ during the pasture period, and from 82.19 to 87.49 cm^2^ during feedlot. The higher REA for Brahman animals reflects their greater potential for muscle deposition, especially under intensive nutritional conditions. This performance can be attributed to the breed’s genetics, which favours the formation of larger cuts, contributing to the commercialization potential of these cuts. A similar relationship has previously been reported in Nellore cattle, where heavier animals tended to present larger ribeye areas relative to live weight [35,36].

Regarding fat thickness, significant variation was observed only for the loin during the pasture period and for the rump during the feedlot period. In general, Brahman cattle exhibited the highest values for these traits; however, Sindhi animals also showed notable fat thickness measurements. Although Sindhi cattle are genetically predisposed to a smaller body frame compared to the other breeds evaluated in this study, they demonstrated greater subcutaneous fat deposition than breeds with higher body growth potential, such as Guzerat. These findings suggest that loin and rump fat thickness values are influenced not only by overall animal development but also by feed conversion (which was higher in Sindhi cattle compared to Brahman and Tabapua; Table 5), thereby contributing to more efficient nutrient conversion into subcutaneous fat.

Regardless of the lower values of REA and fat thickness at loin and rump, observed mainly in Guzerat animals, still exceeded those reported for young uncastrated Nellore bulls fed isoproteic diets with varying soybean content (0% to 24%). For these animals, REA ranged from 70.78 cm^2^ to 75.72 cm^2^, while fat thickness at the loin and rump measured 2.07–2.86 mm and 3.95–4.86 mm, respectively [37]. In general, all breeds showed an increase in the values of rib eye area and fat thickness during the feedlot period compared to the pasture rearing period, which can be attributed to animal development and feed efficiency [35,37]. Therefore, these results are directly related to the data on weight measurements, where the heaviest racial groups and those with better feed efficiency rates resulted in the highest values for muscle development measures assessed by ultrasonography. This behaviour is consistent with previous studies indicating that intensive systems favour the deposition of both muscle and fat tissue, maximizing carcass yield and the quality of cuts.

### 4.3. Carcass

Physiological maturity values indicated characteristics consistent with young animals, with distinct separation of the sacral vertebra and absence of ossification in the lumbar and thoracic vertebrae. The meat exhibited a lean colour classified as light grayish red and a very fine lean texture, further confirming the chronological quality of the animals. Higher values are reported in older animals and may negatively influence meat quality, particularly in attributes such as tenderness and colour, due to increased collagen cross-linking and meat darkening [31,38,39].

Traditionally, Zebu cattle exhibit little to no fat deposition capacity, especially when young or compared to taurine cattle [38,40,41]. Vazquez-Mendoza et al. [38] evaluated the effect of different pure and crossbred breeds of Zebu and taurine cattle in steers and reported a marbling score of 137.1 for Zebu, while the marbling score for Zebu-taurine crosses ranged from 71.2 to 186.9. For pure taurine breeds, the marbling score was 148.6 for European Brown Swiss and 238.1 for Holstein. The marbling content among the different breeds in this study ranged from 112.20 to 136.00, classifying the samples as “Traces” (Standard carcass), according to the USDA Quality Grades [22,23]. This demonstrates the potential of the Zebu breeds used in this study for fat deposition, at a young age, even if in low quantities compared to taurine breeds.

Like marbling, fatness scores showed low differences across the racial groups, reflecting the impact of standardized management during the different animal production periods. Medium fat cover carcasses (fatness score = 3) indicate a fat thickness between 4 and 6 mm, while slight fat cover carcasses (fatness score = 2) had a fat thickness between 1 and 3 mm. For the different breeds evaluated in this study, their carcasses exhibited a fat cover closer to the median (fatness score ranging from 2.65 to 2.76). However, the carcass fat scores were slightly higher than those reported for Zebu bulls aged 18 to 24 months (fatness score < 2.5) [42]. Higher fat cover scores play an essential role in thermal insulation during carcass cooling, preventing muscle shortening and consequently contributing to ensuring a less tough meat [43].

Fat thickness and rib eye area are important metrics related to animal development performance, production yield, profitability, and meat commercialization [44,45]. For example, to export Brazilian meats to Europe, the Hilton Quota requirements must be met, which stipulate carcasses with minimal fat cover from 1 mm to 6 mm, along with other specifications for sex, age, and carcass conformation; carcasses with absent fat cover (<1 mm) are destined for another market [46]. The different racial groups evaluated in this study were within this range, varying between 3.28 mm and 4.45 mm, with values similar to Nellore and other uncastrated Zebu breeds (3.76–5.39 mm) [6] and slightly lower than those of super-precocious Zebu animals of the Canchim (5.00 mm) and Nellore (6.90 mm) breeds [47].

The larger loin eye area in Brahman animals reflects their greater muscle deposition, resulting in heavier live weights and heavier hot carcass weights, reinforcing their aptitude to produce larger cuts. The variation in REA observed in the animals of this study was close to those described for super-precocious Zebu cattle from the Canchim, Nellore, and their crosses, ranging from 67.90 to 91.30 cm^2^ [47], and slightly higher than those previously reported for Brahman (71.0–73.3 cm^2^) [32], and Nellore slaughter at 24 months of age (72.8 cm^2^) [45]. Brahman genetics also positively influenced REA when crossed with Nellore; ½ Brahman × ½ Nellore animals had an REA of 64.1 cm^2^, while ½ Guzerat × ½ Nellore and purebred Nellore had values of 61.3 cm^2^ and 60.5 cm^2^, respectively [31].

The lower hot carcass weight for Sindhi animals (285.9 kg) compared to the other breeds (333.4–356.9 kg) is also a reflection of animal development during the different periods of pasture and confinement feeding. These values were higher than those previously reported in studies evaluating different Zebu breeds under various production and feeding systems [4,5,31,47,48]. They were also similar to or higher than the hot carcass weights reported for taurine animals [33,34,49]. Larger hot carcass weights are advantageous for commercialization as they increase economic efficiency per processed unit.

Even with the second-highest hot carcass weight numerically, Brahman animals had the lowest carcass yield at 54.40%, indicating a higher proportion of non-carcass components, but without strongly impacting the final profitability of the producer or slaughterhouse. This is because their value was similar to those for the other breeds in this study, ranging from 54.40% to 56.78%. These values were close to the 55.75% carcass yield reported for Guzerat animals finished on a diet containing 50 to 80% concentrate [5], but slightly lower than the 56.8–58.2% yield for Brahman cattle slaughtered at 34–35 months of age [32]. The low variation in yield among the animals in this study can be explained by the similar production conditions, such as the same physiological maturity, equal time in pasture and feedlot periods, and identical feeding [47]. Mendonça et al. [40] also reported little variation in carcass yield for cull cows of the Angus, Hereford, Nellore, and Caracu breeds, as well as their crossbred counterparts (46.9–49.9%).

Another production indicator is the total usable meat, which is based on a widely used measure in Brazilian genetic improvement programs as well as in animal production competitions in Brazil. Despite significant influence between the racial groups, the variation in total usable meat was low, ranging from 75.19% to 76.46%, and similar to the 75.33% reported for uncastrated Nellore animals slaughtered at 545.5 kg [50]. Although the Sindhi showed a predisposition for smaller body development, resulting in lower live and carcass weights, they exhibited superior carcass yield and total usable meat content compared to Brahman and Tabapua animals, respectively. This finding highlights the market potential of the evaluated genetic groups, either favoring heavier animals with greater muscle deposition for producers targeting larger cuts, or favoring smaller-framed animals with lower feed intake and reduced production costs, yet capable of delivering high carcass yields and usable meat percentages.

## 5. Conclusions

This study demonstrates the significant impact of genetics and management on the production performance of purebred intact Zebu cattle. Brahman stood out for its superior weight gain, feed efficiency, and muscle deposition, making it highly suitable for markets demanding larger cuts. Moreover, while Zebu beef is traditionally associated with lower marbling and tenderness, our findings highlight the potential of young, intact Zebu cattle for efficient beef production in Brazil, with good muscle growth performance and adequate fat deposition, improving its competitiveness in both domestic and international markets. Thus, targeted genetic and nutritional strategies can further optimize Zebu beef production, aligning with evolving meat markets. These insights contribute to meat science and provide practical guidance for refining breeding and management practices in Brazilian beef production. However, future studies are recommended to assess the economic criteria of production, including cost–benefit analyses, as well as comprehensive evaluations of the overall meat quality of Brahman, Guzerat, Sindhi, and Tabapua animals. Such investigations would complement the present findings by providing a broader perspective on the entire beef supply chain of these Zebu genotypes.

## Figures and Tables

**Table 1:** Description of the EPMURAS method.

Score	Definition		Description	Minimum Score	Maximum Score
	Portuguese	English			
E	Estrutura corporal	Body structure	Measurement of body length and rib depth	1—small	6—large
P	Precocidade	Early maturity	Fat deposition assessment via rib length and limb height ratio	1—late	6—early
M	Musculosidade	Muscularity	Evaluation of muscle mass, especially in the hindquarter and dorsal	1—less	6—more
U	Umbigo	Navel	Assessment of umbilical fold size and placement	1—adhered	6—pendulous
R	Caracterização racial	Racial traits	Head shape, skin color, and breed-specific traits	1—weak	4—excellent
A	Aprumos	Legs	Proportion, angulation, and joint direction of front and rear limbs	1—weak	4—excellent
S	Características sexuais	Sexual characteristics	Development and functionality of external genitalia	1—weak	4—excellent

E = body structure; P = early maturity; M = muscularity; U = navel; R = racial traits; A = legs; S = sexual characteristics.

**Table 2:** Values of visual scores of EPMURAS indicators for young, intact purebred Zebu of different breeds.

Score	Brahman (n = 17)			Guzerat (n = 25)			Sindhi (n = 23)			Tabapua (n = 41)		
	Mean	Min	Max	Mean	Min	Max	Mean	Min	Max	Mean	Min	Max
E	3.9	3.3	4.6	4.1	3.7	4.5	3.7	3.2	4.3	4.1	3.7	4.5
P	3.7	3.1	4.3	3.3	2.9	3.6	4.0	3.4	4.6	3.6	3.2	4.0
M	3.8	3.2	4.5	3.4	3.0	3.7	3.9	3.3	4.6	3.7	3.3	4.1
U	3.8	3.4	4.2	3.0	2.6	3.5	3.9	3.4	4.3	3.5	3.2	3.9
R	2.6	2.2	3.1	3.0	2.8	3.3	2.6	2.2	3.0	3.0	2.7	3.3
A	2.8	2.5	3.1	2.8	2.7	3.0	2.7	2.5	3.0	2.8	2.6	2.9
S	3.5	3.1	3.8	3.3	2.9	3.7	3.0	2.6	3.4	3.5	3.2	3.7

E = body structure; P = early maturity; M = muscularity; U = navel; R = racial traits; A = legs; S = sexual characteristics.

**Table 3:** Composition of feed during the pasture-feeding period.

FEED Type	DM	CP	NDF	ADF	HEM	NFC	TDN
Dry season grass (% DM)	47.13	8.32	70.18	36.85	33.33	13.54	58.97
Rainy season grass (% DM)	24.13	16.86	61.71	29.03	32.68	14.30	62.25
Corn silage (% DM)	33.68	7.29	44.35	30.88	13.47	42.55	65.38
Hay (% DM)	92.73	9.60	70.24	33.14	37.10	9.78	58.96

DM = dry matter; CP = crude protein; NDF = neutral detergent fiber; ADF = acid detergent fiber; HEM = hemicellulose; NFC = non-fibrous carbohydrates; TDN = total digestible nutrients.

**Table 4:** Growth performance of purebred intact male zebu cattle of different breeds at different periods.

Item	Brahman (n = 17)	Guzerat (n = 25)	Sindhi (n = 23)	Tabapua (n = 41)	SEM	*p*-Value
***Suckling period (8–9 months)***						
Birth weight (kg)	35.65 ^a^	34.68 ^a^	27.52 ^b^	34.68 ^a^	0.521	<0.001
Final weight (kg)	223.62 ^ab^	233.16 ^a^	202.39 ^b^	232.44 ^a^	3.196	<0.01
Daily weight gain (kg/day)	0.74 ^ab^	0.78 ^a^	0.68 ^b^	0.78 ^a^	0.011	<0.01
Total gain (kg)	187.97 ^ab^	198.48 ^a^	174.87 ^b^	197.76 ^a^	3.050	<0.05
***Pasture period 1 (140 days) ^1^***						
Initial weight (kg)	223.62 ^ab^	233.16 ^a^	202.39 ^b^	232.44 ^a^	3.196	<0.01
Final weight (kg)	297.82 ^ab^	317.64 ^a^	276.48 ^b^	313.07 ^a^	3.831	<0.001
Daily weight gain (kg/day)	0.53	0.60	0.53	0.58	0.012	0.13
Total gain (kg)	74.21	84.48	74.09	80.63	1.739	0.13
***Pasture period 2 (140 days) ^2^***						
Initial weight (kg)	297.82 ^ab^	317.64 ^a^	276.48 ^b^	313.07 ^a^	3.831	<0.001
Final weight (kg)	409.82 ^a^	422.44 ^a^	359.48 ^b^	422.20 ^a^	4.421	<0.001
Daily weight gain (kg/day)	0.80 ^a^	0.75 ^a^	0.59 ^b^	0.78 ^a^	0.013	<0.001
Total gain (kg)	112.00 ^a^	104.80 ^a^	83.00 ^b^	109.12 ^a^	1.853	<0.001
***Total pasture period (280 days)***						
Initial weight (kg)	223.62 ^ab^	233.16 ^a^	202.39 ^b^	232.44 ^a^	3.196	<0.01
Final weight (kg)	409.82 ^a^	422.44 ^a^	359.48 ^b^	422.20 ^a^	4.421	<0.001
Daily weight gain (kg/day)	0.67 ^a^	0.68 ^a^	0.56 ^b^	0.68 ^a^	0.009	<0.001
Total gain (kg)	186.21 ^a^	189.28 ^a^	157.09 ^b^	189.76 ^a^	2.650	<0.001
***Fattening period (130 days)***						
Initial weight (kg)	409.82 ^a^	422.44 ^a^	359.48 ^b^	422.20 ^a^	4.421	<0.001
Final weight (kg)	628.86 ^a^	602.72 ^a^	508.64 ^b^	622.91 ^a^	6.678	<0.001
Daily weight gain (kg/day)	1.66 ^a^	1.37 ^b^	1.13 ^c^	1.52 ^a^	0.025	<0.001
Total gain (kg)	219.04 ^a^	180.28 ^b^	149.16 ^c^	200.72 ^a^	3.278	<0.001

SEM = standard error of mean. ^1^ Pasture period 1 represents the dry season period. ^2^ Pasture period 2 represents the rainy season period. ^a,b,c^ Values within a row with different superscripts differ significantly at *p* < 0.05.

**Table 5:** Feed intake during the fattening period of purebred uncastrated zebu cattle of different breeds.

Item	Brahman (n = 17)	Guzerat (n = 25)	Sindhi (n = 23)	Tabapua (n = 41)	SEM	*p*-Value
Total water intake (kg/day)	36.41 ^a^	42.15 ^a^	27.89 ^b^	38.17 ^a^	0.710	<0.001
Total feed intake (kg/day)	21.57 ^a^	22.14 ^a^	18.00 ^b^	21.57 ^a^	0.273	<0.001
Dry matter intake (kg/day)	12.19 ^a^	12.51 ^a^	10.17 ^b^	12.19 ^a^	0.155	<0.001
Feed conversion ^1^	7.42 ^b^	9.27 ^a^	9.08 ^a^	8.05 ^b^	0.109	<0.001
Feed efficiency ^2^	0.136 ^a^	0.109 ^c^	0.111 ^c^	0.125 ^b^	0.002	<0.001

SEM = standard error of mean. ^1^ Feed conversion: kg dry matter intake/kg daily weight gain. ^2^ Feed efficiency: kg daily weight gain/kg dry matter intake. ^a,b,c^ Values within a row with different superscripts differ significantly at *p* < 0.05.

**Table 6:** Ultrasound measurements during the grazing and feedlot phases of purebred uncastrated male zebu cattle of different breeds.

Item	Brahman (n = 17)	Guzerat (n = 25)	Sindhi (n = 23)	Tabapua (n = 41)	SEM	*p*-Value
***Pasture period (240 days)***						
Rib eye area (cm^2^)	75.43 ^a^	69.60 ^b^	69.17 ^b^	70.44 ^b^	0.747	<0.05
12th rib fat thickness (mm)	2.38	2.21	2.37	2.37	0.029	0.13
Rump fat thickness (mm)	4.45 ^a^	3.75 ^b^	4.15 ^ab^	3.89 ^b^	0.073	<0.01
***Fattening period (130 days)***						
Rib eye area (cm^2^)	92.31 ^a^	82.19 ^c^	82.46 ^bc^	87.49 ^ab^	0.806	<0.001
12th rib fat thickness (mm)	4.78 ^ab^	4.31 ^b^	4.69 ^ab^	5.22 ^a^	0.098	<0.01
Rump fat thickness (mm)	7.23	6.67	7.03	6.95	0.146	0.63

SEM = standard error of mean. ^a,b,c^ Values within a row with different superscripts differ significantly at *p* < 0.05.

**Table 7:** Hot and cold carcass attributes of purebred uncastrated male zebu cattle of different breeds.

Item	Brahman (n = 17)	Guzerat (n = 25)	Sindhi (n = 23)	Tabapua (n = 41)	SEM	*p*-Value
Skeletal maturity ^1^	145.29 ^b^	156.80 ^b^	174.78 ^a^	160.49 ^b^	1.845	<0.001
Lean maturity ^1^	185.29 ^b^	226.00 ^a^	189.13 ^b^	207.32 ^ab^	4.452	<0.01
Marbling ^2^	117.65	136.00	130.43	112.20	4.084	0.11
Fatness scores	2.65	2.76	2.74	2.68	0.044	0.84
Hot carcass weight (kg)	342.09 ^a^	333.36 ^a^	285.85 ^b^	353.88 ^a^	3.992	<0.001
Carcass yield (%)	54.40 ^c^	55.29 ^bc^	56.11 ^ab^	56.78 ^a^	0.166	<0.001
Total usable meat (%)	76.46 ^a^	75.69 ^ab^	76.23 ^a^	75.19 ^b^	0.138	<0.01
12th rib fat thickness (mm)	3.79 ^ab^	3.28 ^b^	3.63 ^ab^	4.45 ^a^	0.170	<0.05
Rib eye area (cm^2^)	85.35 ^a^	75.34 ^ab^	73.28 ^b^	79.33 ^ab^	0.957	<0.01

SEM = standard error of mean. ^1^ USDA scores, ranging from 100 (A00) to 500 (E00). ^2^ USDA marbling scores, ranging from Practically Devoid (0–100) to Traces (101–200). ^a,b,c^ Values within a row with different superscripts differ significantly at *p* < 0.05.

## Data Availability

The raw data supporting the conclusions of this article will be made available by the authors on request.

## Supplementary Materials

The following supporting information can be downloaded at: https://www.mdpi.com/article/10.3390/ani15203024/s1, Table S1: Composition of the protein-energy supplement used in the animals’ diet during the pasture-feeding period (composition provided by the producer).

## Abbreviations

The following abbreviations are used in this manuscript:

E	Body structure
P	Early maturity
M	Muscularity
U	Navel
R	Racial traits
A	Legs
S	Sexual characteristics
N	Nitrogen
P_2_O_5_	Phosphorus pentoxide
K_2_O	Potassium oxide
CP	Crude protein
TDN	Total digestible nutrients
DM	Dry matter
NDF	Neutral detergent fiber
ADF	Acid detergent fiber
HEM	Hemicellulose
NFC	Non-fibrous carbohydrates
REA	Rib eye area
USDA	United States Department of Agriculture
HCW	Hot carcass weight
LW	Live weight
SFT	Subcutaneous fat thickness
ANOVA	Analysis of variance
SEM	Standard error of mean
