# Supplementary material for: The Impact of Purebred Zebu Breeds on Growth Performance and Carcass Characteristics
Source: Animals (Basel). 2025 Oct 17;15(20):3024. doi: 10.3390/ani15203024 (PMC12562142; doi:10.3390/ani15203024)
Supplement: Supplementary file 1 [file animals-15-03024-s001.zip › animals-3921479-supplementary.pdf]

Supplementary Table S1. Composition of the protein-energy supplement used in the animals' diet during the pasture-feeding period (composition provided by the producer).

Component	Unit of measure	Dry season	Rainy season
Linoleic acid (min.)	mg/kg	8.05	8.05
Oleic acid (min.)	mg/kg	6.83	6.83
Calcium (max.)	g/kg	20.00	20.00
Calcium (min.)	g/kg	10.00	10.00
Cobalt (min.)	mg/kg	7.5	15.00
Copper (min.)	mg/kg	40.00	144.00
Choline (min.)	mg/kg	160.00	160.00
Organic chromium (min.)	mg/kg	0.20	0.20
Sulfur (min.)	mg/kg	3,000.00	6,000.00
Ether extract (min.)	g/kg	10.00	15.00
Factor P (min.)	mg/kg	4,000.00	4,000.00
Fluorine (min.)	mg/kg	50.00	60.00
Phosphorus (min.)	mg/kg	5,000.00	6,000.00
Iodine (min.)	mg/kg	5.00	12.00
Lysine (min.)	mg/kg	20.00	20.00
Mannanoligosaccharides (min.)	mg/kg	24.00	24.00
Methionine (min.)	mg/kg	1.60	1.60
TDN – Total Digestible Nutrients (min.)	g/kg	620.00	650.00
NPN – protein equivalent (max.)	g/kg	118.00	22.00
Omega 3 (min.)	mg/kg	1.18	1.18
Omega 6 (min.)	mg/kg	8.05	8.05
Omega 9 (min.)	mg/kg	7.52	7.52
Crude protein (min.)	g/kg	240.00	120.00
Saccharomyces cerevisiae (min.)	CFU/kg	4.00×10^8	4.00×10^8
Selenium (min.)	mg/kg	0.90	0.90
Sodium (min.)	g/kg	22.00	22.00
Tyrosine (min.)	mg/kg	4.00	4.00
Zinc (min.)	mg/kg	140.00	250.00
Organic zinc (min.)	mg/kg	4.20	4.40

CFU = colony-forming units.
